# *Agrimonia eupatoria* L. and *Cynara cardunculus* L. Water Infusions: Phenolic Profile and Comparison of Antioxidant Activities

**DOI:** 10.3390/molecules201119715

**Published:** 2015-11-18

**Authors:** Anika Kuczmannová, Peter Gál, Lenka Varinská, Jakub Treml, Ivan Kováč, Martin Novotný, Tomáš Vasilenko, Stefano Dall’Acqua, Milan Nagy, Pavel Mučaji

**Affiliations:** 1 Department of Pharmacognosy and Botany, Faculty of Pharmacy, Comenius University, Odbojárov 10, 832 32 Bratislava, Slovakia; kuczmannova@fpharm.uniba.com (A.K.); nagy@fpharm.uniba.sk (M.N.); 2 Department of Pharmacology, Faculty of Medicine, Pavol Jozef Šafárik University, Trieda SNP 1, 040 11 Košice, Slovakia; lenka.varinska@upjs.sk; 3 Department for Biomedical Research, East-Slovak Institute of Cardiovascular Diseases, Inc., Ondavská 8, 040 11 Košice, Slovakia; ivankovac.kovi@gmail.com (I.K.); martin.novotny.dr@gmail.com (M.N.); tomasvasilenko@gmail.com (T.V.); 4 Institute of Anatomy, 1st Faculty of Medicine, Charles University, U nemocnice 2, 128 00 Prague, Czech Republic; 5 Department of Molecular Biology and Pharmaceutical Biotechnology, Faculty of Pharmacy, University of Veterinary and Pharmaceutical Sciences, Palackého 1-3, 612 42 Brno, Czech Republic; tremlj@vfu.cz; 6 2nd Department of Surgery, Pavol Jozef Šafárik University and Louise Pasteur University Hospital, 041 90 Košice, Slovakia; 7 Department of Infectology and Travel Medicine, Pavol Jozef Šafárik University and Louise Pasteur University Hospital, 041 90 Košice, Slovakia; 8 Department of Surgery, Pavol Jozef Šafárik University and Košice-Šaca Hospital, 040 15 Košice-Šaca, Slovakia; 9 Department of Pharmaceutical and Pharmacological Sciences, University of Padova, Via F. Marzolo 5, 351 31 Padova, Italy; stefano.dallacqua@unipd.it

**Keywords:** ROS, oxidative stress, agrimony, artichoke, antioxidants

## Abstract

Reactive oxygen species (ROS) are highly considered in the ethiopathogenesis of different pathological conditions because they may cause significant damage to cells and tissues. In this paper, we focused on potential antioxidant properties of two medical plants such as the *Agrimonia eupatoria* L. and *Cynara cardunculus* L. Both plants have previously been studied for their pharmacological activities, especially as hepatoprotective and hypoglycemic activities. It has been suggested, that their effects are related to the antioxidant properties of polyphenols, which are dominant compounds of the plants’ extracts. In the present study HPLC-MS analysis of water infusion was performed allowing the identification of several phenolic constituents. Furthermore, antioxidant effects of the two extracts were compared showing higher effects for agrimony extract compared to artichoke. Thus, agrimony was selected for the *in vivo* study using the skin flap viability model. In conclusion, our results provide evidence that the *A. eupatoria* extract may be a valuable source of polyphenols to be studied for the future development of supplements useful in the prevention of diseases linked to oxidative stress.

## 1. Introduction

The plant kingdom is considered one of the main sources of drugs, as demonstrated by several examples, but in the recent years the growing use of herbal medicine and food supplements containing plant extracts has been recorded. Accordingly, it is estimated that almost 80% of the world’s population uses medicine of herbal origin. In the last few decades, there has been increasing interest in the potential health benefits of several plant extracts and many of the health-related effects are linked to the presence of the secondary metabolites, such as the polyphenols. These compounds may reduce the risk of the development of several diseases due to a complex effect but many authors claim that some of their properties may be related both to their antioxidant capacity and other biological activities [1]. Flavonoids belong to the group of polyphenols, and are considered at least in part responsible for the biological properties of several medicinal plants, e.g., anti-inflammatory, antiviral, antibacterial, neuroprotective, anti-ulcerogenic, antispasmodic, anti-thrombotic, anti-cancer, and others [2,3]. Previous findings also correlated the biological activities of flavonoids with their antioxidant effects due to the intimate relation between ROS and inflammation, thus resulting in specific structure moieties (such as catechol group) which allow scavenging of free radicals [2,3].

Oxidative stress is defined as an imbalance between the production of free radicals, on the one hand, and antioxidant defense, on the other hand [4]. Such stress frequently occurs during aging, cancer, cardiovascular disease, cataracts, immune system decline, brain dysfunction, diabetic vasculopathy, *etc.* and may lead to cell and/or tissue damage [5,6,7]. It is characterized by enhanced production of reactive oxygen species (ROS), which results in damage of cellular proteins and lipids, decreased production of nitric oxide (NO), activation of different transcription factors (AP-1, NF-κB), as well as increased levels of pro-inflammatory cytokines (IL-1, IL-6, TNF-α) and mediators (ICAM-1) [6,7,8]. Some cells and tissues exhibit higher sensitivity to oxidative stress and ROS due to low levels of antioxidant enzymes, such as the catalase (CAT), superoxid dismutase (SOD), and glutathion peroxidase (GPx) [9].

In this study, we considered two different medicinal plants, agrimony (*Agrimonia eupatoria* L.) and artichoke (*Cynara cardunculus* L.) as potential antioxidant agents. Such plants are well known and used for their medicinal purposes and present low toxicity. Previous investigations revealed that both plants contain polyphenols which, in numerous publications, were referred as the active substances responsible for its protective effects. In this context, it has been reported that many plants that contain polyphenols induce endothelium-dependent vasorelaxation via NO release and/or increase of NO biological activity [10]. Previously, it has been shown that agrimony and artichoke demonstrate significant antioxidant properties by means of free radical scavenging capacity [2,11,12,13,14,15,16,17,18,19,20]. These plants are listed in many papers as herbs with various beneficial effects. For instance, agrimony water extracts exhibit “insulin-like” activities based on the stimulation of pancreatic β-cells and inhibition of α-glucosidase [12,21,22], as well as exhibits hepatoprotective [23] and neuroprotective effects [24] based on its antioxidant activities. Furthermore, it has been demonstrated that artichoke extracts decrease the postprandial glucose levels in diabetic rats [25,26,27], and also exert anti-hyperlipidemic activities [25,26,28,29].

Therefore, the main goal of our study was to explore the antioxidant properties of the above mentioned plant extracts in a series of *in vitro* and *in vivo* experiments in an attempt to find new therapeutic agents supporting current treatment of various diseases linked to oxidative stress. As bioactivity of extract is strongly influenced by phytochemical composition accurate HPLC-MS analysis was carried on in order to obtain a qualitative/quantitative profile of the tested material.

## 2. Results and Discussion

### 2.1. Extraction and Extract Composition

Selection of the extracting solvent is an important part of the experiment as it affects extract composition and properties [30]. Alcohol and alcohol-water mixtures have been considered to be the most efficient solvents for low-molecular weight polyphenols which are characterized by high antioxidant activity [31]. However, in the present experiment lyophilized water extracts were used. The preparation of such an extract is similar to the preparation of an herbal tea and, thus, presents a safe, simple, and efficient way of extraction [31,32].

Phytochemical content of the two extracts was analyzed by HPLC-MS leading to the identification and quantification of the main phenolic constituents based mainly on fragmentation patterns or by comparison with reference compounds. The HPLC-MS chromatogram of the artichoke dried extract presented several peaks that can be related to caffeoyl quinic acid derivatives, as well as luteolin and apigenin glycosides. The most abundant compounds are 4-caffeoylquinic acid, luteolin-7-*O*-glucoside, and the 3,5-dicaffeoyl quinic acid (Table 1a). The agrimony extract presented also several phenolic constituents mainly apigenin, kaempferol, and quercetin derivatives, as well as catechin and oligomeric proantocyanidins (Table 1b). The most abundant compounds are quercetin glycosides and proantocyanidin trimers. Both lyophilized extracts presented a total amount of polyphenols of about 8% being a significant source of such phytoconstituents.

Previously, it has been detected that the artichoke extract contains polyphenols in the range from 5.3 to 13.3 g/kg of dry matter [33] and, thus, similar to data obtained during the present investigation. However, substantial differences were found in the composition of the phenolic content. As mentioned above, the abundant compounds are 4-caffeoylquinic acid, luteolin-7-*O*-glucoside, and 3,5-dicaffeoyl quinic acid, but these caffeoyl quinic acids were not detected in the alcoholic extract studied by Pandino and coworkers. Furthermore, another study showed that the agrimony water extract contains 8.2–10.9 mg/g flavonoids, 6.3–10.9 mg/g of tannins, and 0.6–0.9 mg/g phenolic acids [34] which is similar to data presented in this current investigation.

**Table 1:** Identification of constituents and quantification from *Cynara cardunculus* (**a**) and *Agrimonia* *eupatoria* (**b**).

Identification	Retention Time (Rt) (min)	[M − H]^−^	Fragments	% *w*/*w*
**(a) *Cynara cardunculus***				
Quinic acid *	1.5	191		0.615 ± 0.01
1-caffeoyl quinic acid	3.15	353	191, 179, 85	0.137 ± 0.01
3-caffeoyl quinic acid *	5.2	353	191, 179, 135, 85	0.133 ± 0.01
4-caffeoyl quinic acid	5.7	353	191, 173, 135, 93	1.646 ± 0.04
5-caffeoyl quinic acid *	7	353	191,179, 135	0.320 ± 0.02
Luteolin-7-*O*-glucoside *	11	447	285, 267, 241, 217	1.603 ± 0.03
Luteolin-7-*O*-glucuronide *	11.6	461	285, 267, 241, 217	0.167 ± 0.02
Luteolin-7-*O*-acetylglucoside	13.5	489	285, 267, 241, 217	0.594 ± 0.02
Caffeoil-hexoside	2.1	341	179	0.093 ± 0.01
Luteolin-7-*O*-rutinoside *	10.3	593	285, 267, 241, 217	0.086 ± 0.01
Apigenin-7-*O*-glucoside *	13.1	431	269, 241, 225	0.131 ± 0.01
Apigenin-7-*O*-rutinoside	12.2	577	269, 241, 225	0.017 ± 0.01
1,3-dicaffeoyl quinic acid *	4.3	515	353, 191, 179	0.083 ± 0.01
1,4-dicaffeoyl quinic acid *	7.9	515	353, 179, 173	0.042 ± 0.01
3,4-dicaffeoyl quinic acid	12	515	353, 299, 203, 179	0.189 ± 0.01
3,5-dicaffeoyl quinic acid *	12.5	515	353, 203, 191, 179	1.823 ± 0.01
4,5-dicaffeoyl quinic acid	13.7	515	353	0.268 ± 0.03
**Total amount**				**7.85**
**(b) *Agrimonia eupatoria***				
Quinic acid *	1.5	191	111, 57	0.360 ± 0.01
*p*-coumaric acid *	4	163		1.330 ± 0.01
Catechin *	13	289	245, 205, 175	0.200 ± 0.01
Quercitin-acetil-glucoside	9	505	445, 301, 271, 255, 179, 151	0.670 ± 0.02
Rutin *	11.0	609	301, 271, 255, 179, 151	0.155 ± 0.01
Apigenin derivative	12.5	447	307, 269	0.200 ± 0.01
*p*-Coumaroil quinic acid	8	337	163, 191	0.260 ± 0.01
5-caffeoyl quinic acid *	7.1	353	191	0.510 ± 0.01
Luteolin-7-*O*-glucuronide *	12.5	461	285,257, 229	0.270 ± 0.01
Caffeoil-hexoside	2.1	341	179	0.100 ± 0.01
Kaempferol-*p*-coumaroyl-hexoside	15.6	593	285	0.180 ± 0.01
Quercetin-acetyl-hexoside	6.7	505	301	0.730 ± 0.01
Procyanidin B-1 *	27.0	577	425, 407, 289	0.180 ± 0.01
Procyanidin B-3	29	577	425, 407, 289	0.140 ± 0.01
Procyanidin-trimer-B	8	865	695, 577, 407	0.870 ± 0.01
Procyanidin tetramer-B	15	1153	695, 577, 407	0.300 ± 0.02
Quercetin3-*O*-glucoside *	9.5	463	301, 271, 255, 179, 151	0.550 ±0.009
Quercetin-3-*O*-rhamnoside *	10.1	447	301, 271, 255, 179, 151	0.301 ± 0.01
Quercetin-7-*O*-rhamnoside *	11.2	447	301, 271, 255, 179, 151	0.260 ± 0.01
Apigenin-7-*O*-glucuronide *	11.8	445	269	0.190 ± 0.009
luteolin-acetyl-hexoside	10.5	489	447, 285	0.110 ± 0.009
**Total amount**				**7.87**

Compounds with * were compared with standard.

### 2.2. Cytotoxicity Test

**Figure 1:**
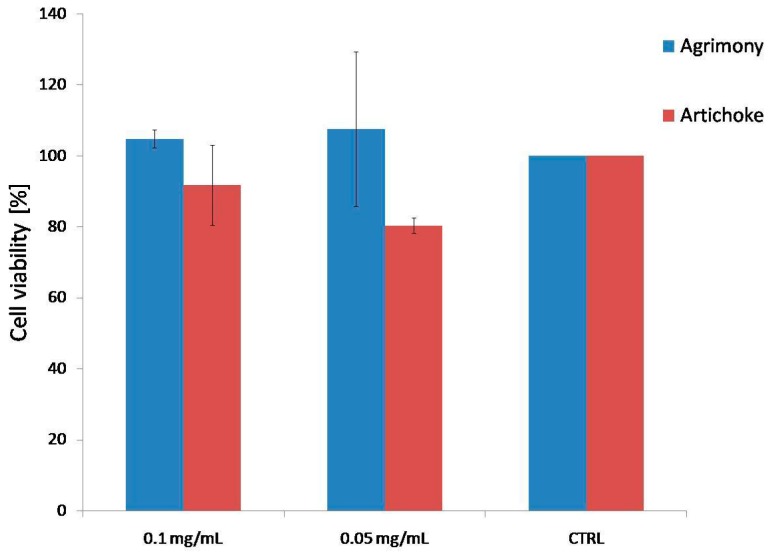
Effect of agrimony and artichoke (0.1 mg/mL and 0.05 mg/mL) on cell viability.

No cytotoxic effect was observed for the water extracts of agrimony (AE) and artichoke (CC) at the tested concentration (0.1 mg/mL and 0.05 mg/mL) on THP-1 cell line after 24 h incubation (Figure 1).

### 2.3. CAT and SOD Levels

**Figure 2:**
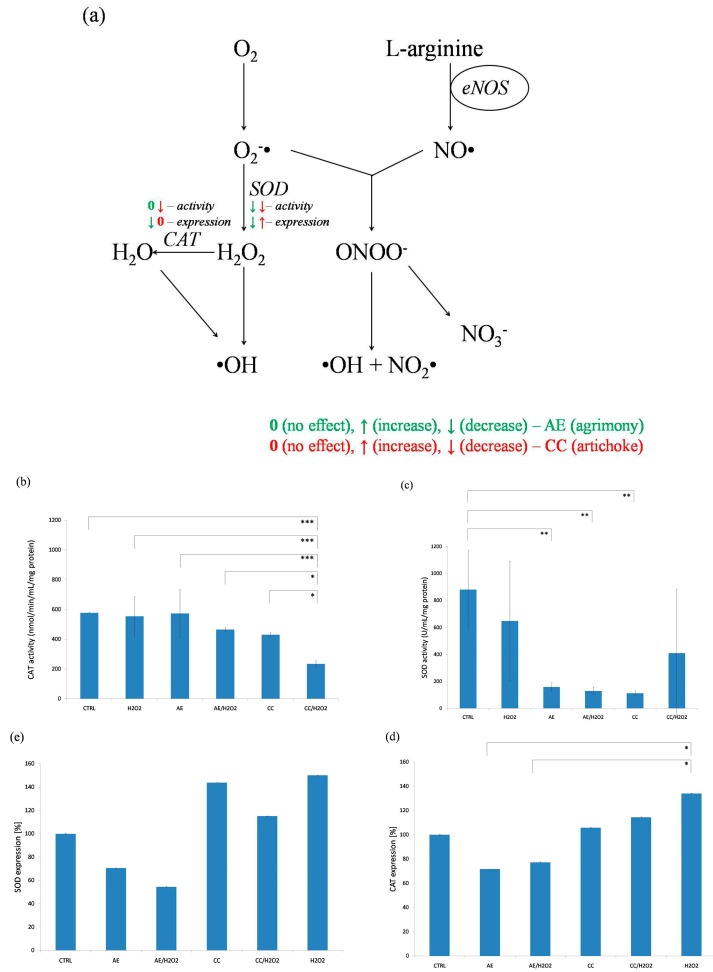
(**a**) Scheme of plant’s effects on NO degradation by superoxide (0—no effect, ↑—increased activity/expression, ↓—decreased activity/expression; AE—green, CC—red); (**b**–**e**) effect of agrimony and artichoke on activity of CAT (**b**) and SOD (**c**) and expression of CAT (**d**) and SOD (**e**) (*** *p* < 0.001, ** *p* < 0.01, * *p* < 0.05).

Oxidative stress is commonly initiated by the overproduction of superoxide anion (O_2_^−^) and hydrogen peroxide (H_2_O_2_). These molecules convert to potent oxidants, such as hydroxyl radical, hypochlorous acid, and peroxynitrate. Because of that, antioxidant enzymes, such as SOD and CAT are candidates for augmentation in antioxidant defense [35]. SOD converts O_2_^−^ into H_2_O_2_ and CAT converts H_2_O_2_ into water (Figure 2a).

The main disadvantage of these enzymes is a relative quick elimination from the bloodstream, which compromises their delivery to the site of biological activity [35]. Based on these data the question of whether water infusions of agrimony and artichoke stimulate the activity and/or expression of SOD and CAT was answered.

It is known that SOD competes with NO for superoxide and reduces the formation of peroxynitrite (Figure 2a) [35,36]. Theoretically, when the interception and degradation of O_2_^−^ and H_2_O_2_ by these enzymes is increased, the degradation of NO should be decreased. Surprisingly, obtained data demonstrate that the AE extract did not alter CAT activity and decreased activity of SOD (Figure 2b,c). Similarly, CC extract had inhibitory effect on both CAT and SOD activities (Figure 2b,c). As a positive control to complete the panel of experiments H_2_O_2_ was added (Figure 2b,c).

Following the activity experiments the expression of CAT and SOD was evaluated as well (Figure 2d,e). Our results showed that AE decreased both the SOD and CAT expressions (Figure 2d,e). Contrarily, the artichoke extract slightly increased the levels of both tested enzymes (Figure 2d,e). On the other hand, extracts in combination with H_2_O_2_ showed increased expression for CAT as well as for SOD (Figure 2d,e). Significant changes were observed only at expression of CAT influenced by artichoke, compared to H_2_O_2_ (Figure 2d).

### 2.4. ABTS Assay

Another possible way to test antioxidant activity is the determination of antioxidant capacity. In this study the scavenging capacity was determined by the ABTS method. According to our results both tested extracts showed good antioxidant properties (RC_50_s were determined as AE = 0.79 mg/mL, CC = 3.73 mg/mL) compared to other studies [14,16,17,37,38]. Based on observed results we may conclude that AE and CC extracts scavenge free radicals (superoxide and hydrogen peroxide radicals) similarly to SOD and/or CAT. CAT and SOD have high affinities for the reaction with reactive oxygen species [35]. The reaction, activity of CAT with hydrogen peroxide depends entirely on the concentration of H_2_O_2_ [39]. According to this, it may be considered that CAT and SOD are more effective against acute and/or massive oxidative stress [35]. This may also be the reason that the expressions of SOD and CAT were higher, but their activities remained lower. Therefore, it may be hypothesized that these enzymes, although present at increased levels, showed lower activities due to radicals scavenged by the plants’ compounds.

### 2.5. Oxidative Damage of Plasmid DNA

**Figure 3:**
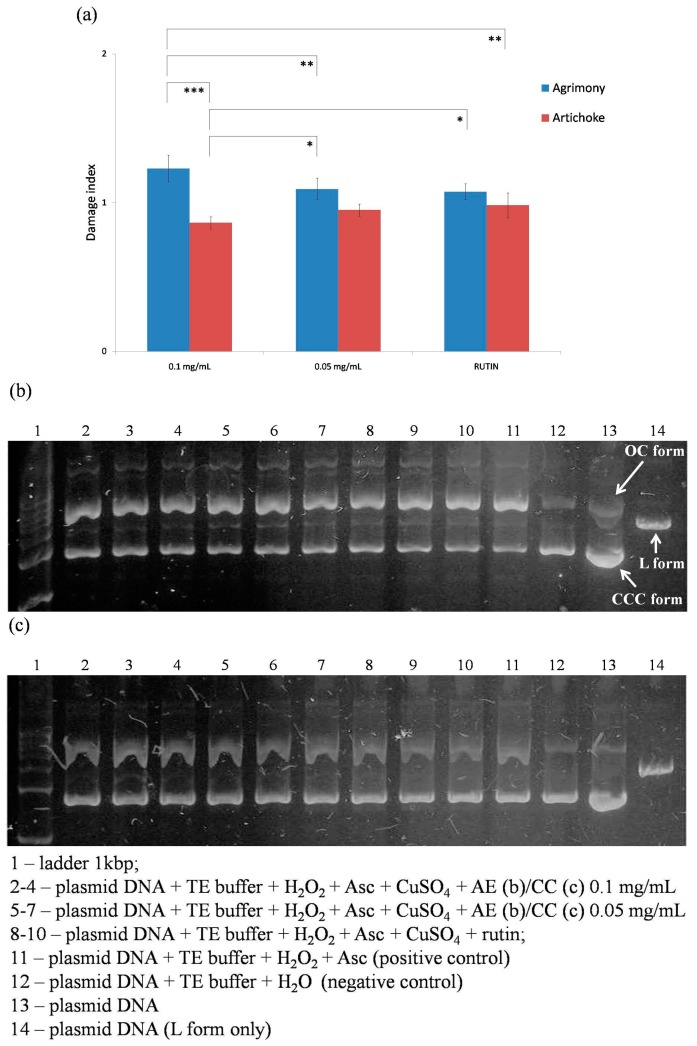
(**a**) Damage indexes of 0.1 mg/mL and 0.05 mg/mL agrimony and artichoke (*** *p* < 0.001, ** *p* < 0.01, * *p* < 0.05); (**b**) gel electrophoresis: agrimony (0.1 mg/mL and 0.05 mg/mL); and (**c**) gel electrophoresis: artichoke (0.1 mg/mL and 0.05 mg/mL).

Protective effects of agrimony and artichoke on oxidative damage of plasmid DNA were evaluated in the next part of our study. The hydroxyl radical is generated from hydrogen peroxide in the presence of trace amounts of metal ions and degrades deoxyribose into fragments [11,40]. The hydroxyl radical causes base oxidation; if the damage is not repaired, DNA alterations lead to mutations and/or cell death [40]. The plasmid DNA consists of three forms, CCC—covalently closed circular (native, not damaged), OC—open circular (the first step of degradation), and L form—linear form (the result of oxidative damage). All forms are visible after electrophoresis under UV light. The OC form is the slowest conformation in the gel and it thus occurs at the top of the gel. The next band is the L form (linear), followed by the CCC form [40]. The results were expressed as damage index—Di. Plasmid DNA was separated in the gel as shown in Figure 3. Water was used instead of the extracts as positive control. The negative control contained only water and TE buffer instead of the Fenton solution. Statistical comparison between damage indexes indicate that agrimony (AE) possesses better antioxidant properties related to the plasmid DNA protection when compared to artichoke (CC) and/or rutin (used as standard) (Figure 3).

### 2.6. Skin Flap Viability

**Figure 4:**
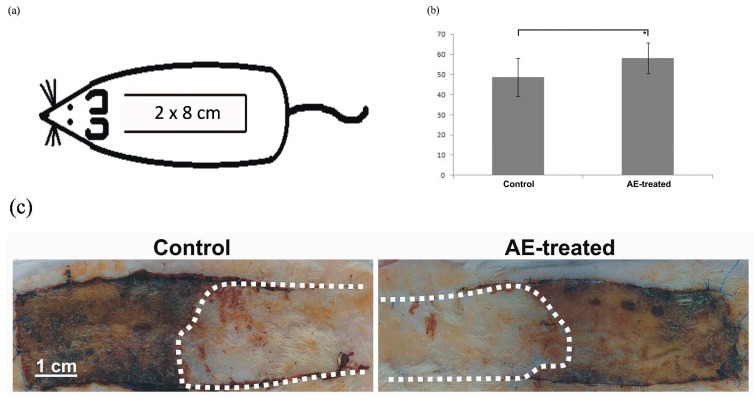
Skin flap in rats following *Agrimonia eupatoria* L. treatment; (**a**) dimensions of the skin flap located on the back of each rat s; (**b**) effect of *A. eupatoria* on skin flap viability expressed in percentages (* *p* < 0.05); and (**c**) photograph showing skin flaps of control and treated rats seven days after surgery (the dotted line labels vital part of the flap).

Our *in vitro* results demonstrate that AE extract showed more efficient antioxidant activity than the CC extract. Hence, this extract was selected for the animal study. The mean percentages of flap viabilities of both groups from different groups can be seen in the Figure 4b. Mean vital area of a skin flap in the control group was 48.7% ± 9.4%, whereas the AE treated rats had increased flap viability to 58.1% ± 7.7% (*p* < 0.05). Several mechanisms might be involved into the protective effect of AE on skin ischemia.

Similarly, we showed in our previous study that estrogen replacement therapy increases skin flap viability in rats [41] by increasing VEGF expression and NO synthesis [42]. Furthermore, increased tissue survival may be related to iNOS-dependent enhancement of VEGF levels and resulting angiogenic response [43]. Since in our study was shown that AE extract demonstrate well the antioxidant, anti-inflammatory, and vasodilatory properties [44], which may be the molecular mechanisms of its protective effect on skin ischemia.

## 3. Experimental Section

Lyophilized water infusions of two commercially-available medicinal plants (Fytopharma, Malacky, Slovak Republic), *Agrimonia eupatoria* L. (agrimony/AE) from the Rosaceae family and *Cynara cardunculus* L. (artichoke/CC) from the Asteraceae family, were used. The water infusions were prepared according to the Pharmacopoeia Bohemoslovaca 4 [45] and the lyophilization (freeze-drying, −53 °C, 0.043 Pa) according to instructions of use (SCANVAC CoolSafe™, LaboGene™, Lynge, Denmark).

### 3.1. High-Performance Liquid Chromatography-Mass Spectrometry (HPLC-MS)

Quali-quantitative analysis of phenolic derivatives in the extract was obtained by HPLC-DAD-MS. The measurements were performed with an Agilent 1260 chromatograph (Santa Clara, CA, USA) equipped 1260 diode array and MS-500 ion trap as detectors. MS spectra were recorded in negative mode in the range of 50–2000 Da. Fragmentation of the main ionic species were obtained by the turbo data depending scanning (TDDS) function. Identification of compounds was achieved on the basis of the fragmentation spectra as well as the comparison with the literature and injection of reference compounds. The ESI ion source was used for analysis. An Agilent Eclipse XDB C-18 (2.1 × 150 mm) 3.5 μm was used as stationary phase. A DAD detector was used quantification of flavonoids and caffeoyl quinic acid derivatives 350 and 330 nm wavelengths were used. The mobile phases were solvent A (water 0.1% formic acid) and solvent B (Acetonitrile). The elution gradient started at 90% A then decreased to 0% over 25 min. Identification of constituents was achieved on the basis of MSn fragmentation experiments (multiple stages of fragmentation/ionization involved), comparison of the obtained fragmentation pathways with the literature and comparison with previously-isolated constituents used as reference compounds of the Natural Product Lab collection. Quantification of phenolic constituents was obtained with the method of calibration curve: rutin (Sigma Aldrich, St. Louis, MO, USA) was used as external standard for flavonoid quantification chlorogenic acid (Sigma Aldrich) was used for caffeoylquinic acid derivatives, catechin (Phytolab, Vestenbergsgreuth, Germany) was used for catechin and procyanidin derivatives in the range 0.5–100 μγ/mL at four different concentration. Calibration curves were Y = 144232X + 112 (R^2^ = 0.9998) for rutin and Y = 194232X + 77 (R^2^ = 0.9999) for cholorgenic acid and Y = 112543X + 48 for catechin respectively.

### 3.2. Human Monocytic Leukemia Cell Line (THP1)

THP-1 cells (ECACC, Salisbury, UK) were cultivated at 37 °C in a humidified air atmosphere containing 5% CO_2_/95% air in RPMI 1640 medium, supplemented with 2 mML-glutamine, 10% FBS, 100 U/mL of penicillin, and 100 μg/mL of streptomycin.

### 3.3. Cyotoxicity Test

Cell viability was measured with WST-1 test according to the manufacturer’s manual. THP-1 cells were seeded in a 96-well plate in triplicate (500,000 cell/mL) and cultivated in a RPMI 1640 serum-free medium at 37 °C. Water extracts of agrimony and artichoke at concentrations of 0.1 mg/mL or 0.05 mg/mL were added to the cells (preliminary testing included following concentrations: 0.1, 0.05, 0.025, 0.0125, and 0.00625 mg/mL). After 24 h the absorbance was measured. The created formazan correlated with the number of metabolically-active cells.

### 3.4. CAT and SOD Activity and Expression

For this part of our experiment cultures THP-1 monocytes were used. These cells were incubated either with agrimony or with artichoke sample at the concentration of 0.1 mg/mL or plant sample supplemented with H_2_O_2_ for 5 h. After the incubation cell lysates were used. The activities of CAT and SOD were determined using assay kits according to the manufacturer instructions (Cayman Chemical Company, Ann Arbor, MI, USA) and their expressions by Western blot analysis. Briefly, proteins were separated using gel electrophoresis (SDS-PAGE), and then transferred to PVDF membrane (semi-dry electroblotting). Briefly, there was blocking of non-specific binding by placing the membrane in non-fat dry milk. Then the membrane was incubated overnight with the primary antibodies, which concentrations were anti-CAT, anti-SOD 1:1000 (Sigma Aldrich). After quadruple washing, the membrane was exposed to secondary antibodies goat-anti-rabbit 1:2000 and goat-anti-mouse 1:2000 (Sigma Aldrich), and incubated for one hour at room temperature. Finally, Opti-4CN Substrate kit (BIO-RAD, Hercules, CA, USA) was used for colorimetric detection.

### 3.5. ABTS Assay

For the determination of scavenging capacity ABTS assay was employed. ABTS assay was performed according to Giăo *et al.* (2012) [16]. The ABTS radical solution was prepared via addition, at 1:1 (*v*/*v*), of ABTS (7 mM) and potassium persulphate (2.45 mM) solutions. The reaction took place in the dark for 24 h. 1.1 mL ABTS solution was diluted with ethanol to 50 mL. For analysis, 20 μL of each extract was mixed with 2 mL of ABTS solution and used to obtain an inhibition by 6 min of reaction. All reactions for each extract and each concentration were carried out in triplicates. Finally, absorbance was measured by spectrophotometer (Genesys 6, Thermo, Electro Corp., Loughborough, UK) at 734 nm.

### 3.6. Determination of Antioxidant Activity Using Oxidative Damage of Plasmid DNA

The pUC19 plasmid DNA used during this experiment was isolated from *Escherichia coli* TOP 10F′ strain. This plasmid was isolated by using the QIAprep Spin Miniprep kit (Qiagen, Hilden, Germany) according to manufacturer’s instructions. The purity of the plasmid DNA was assessed by using the BioPhotometer spectrophotometer (Eppendorf, Hamburg, Germany). The reaction was performed in PCR tubes. Briefly, the reaction was carried out with 300 ng of plasmid DNA in TE buffer. Firstly, agrimony or artichoke sample (at concentrations of 0.1 mg/mL or 0.05 mg/mL), then rutin (100 μM), and finally the Fenton solution consisting of H_2_O_2_ (660 μM), ascorbate (830 μM), and CuSO_4_ 5H_2_O (82.5 μM) were added into the PCR tubes. The reaction mixture was incubated at 37 °C for 1 h. The samples were analyzed using gel electrophoresis separation on 0.8% agarose penetrated with GelRed™ dye for visualization. All reactions were carried out in triplicate. The gel was analyzed under UV light and intensities of each band were measured and expressed as a percentage of the area under the curve (AUC). Equation (1), expressing the amount of intact CCC DNA in each sample divided by the amount of CCC DNA in the negative control sample was calculated. The lowest ratio is expected for positive control samples, because Fenton reagents can damage DNA and no extract is present for protection. The more active antioxidants are present in the tested samples, the higher their ratio should reach. To cover the possible influence of solvent on the reaction, the damage index, dividing the ratio of the sample by the ratio of the positive control (Equation (2)), was calculated. Both plant extracts used were compared to rutin, as a positive control, which is a significant antioxidant agent [40].

ratio = AUC_CCC sample_/AUC_CCC NC_ (1)

damage index = ratio sample/ratio PC(2)

### 3.7. In Vivo Skin Flap Viability Model

The animal experiment was approved by the Ethical Committee of Faculty of Medicine, Pavol Jozef Šafárik University (no. EK-13/2013). Male Sprague-Dawley rats (*n* = 20), six months of age, were used in this study. These were at the beginning of the experiment randomly divided into two groups. Both groups had free access to standard laboratory diet. Control rats had free access to tap water, while the *A. eupatoria*-treated rats had free access only to AE water extract for five weeks prior to the flap surgery.

Flap surgery was performed under general anesthesia which was induced by the intramuscular administration of ketamine (40 mg/kg; Calypsol, Richter Gedeon, Budapest, Hungary), xylazine (15 mg/kg; Rometar a.u.v., Spofa, Prague, Czech Republic), tramadol (5 mg/kg; Tramadol-K, Krka, Novo Mesto, Slovenia), and atropine (0.05 mg/kg; Atropin, Biotika, Slovenská Ľupča, Slovak). Under standard aseptic conditions, a cranially-based over-dimensioned random-pattern skin flap measuring 2 × 8 cm was dissected free from the underlying fascia on the back of each animal (Figure 4a). The flap was immediately returned to their bed and fixed into its original position by an intradermal running suture (Chiraflon 5/0, Chirmax, Prague, Czech Republic).

#### 3.7.1. Macroscopic Measurement of Survived Area on the Skin Flap

All animals were sacrificed seven days after flap surgery and obtained samples were processed for the macroscopic measurement of vital flap area. The flap survival was measured from standardized photographs as follows. Flaps were photographed with a scale immediately after surgery and at day seven using an Olympus E330 digital camera equipped with ED 50 mm f 2.0.

#### 3.7.2. Histological Examination

All skin flaps were firstly fixed for 48 h in 4% buffered formaldehyde, then cut into two 4 cm long specimens (Figure 4c), and routinely processed for light microscopy (dehydration using increasing concentration of ethanol, paraffin embedding, sectioning (7 μm thick), and staining with hematoxylin-eosin).

### 3.8. Statistical Analysis

Data from the *in vitro* and *in vivo* experiments are present as mean ± standard deviation (SD). One way analysis of variance (ANOVA) followed by Tukey-Kramer *post hoc* test were used to compare the differences between groups. Significance was accepted at *p* < 0.05.

## 4. Conclusions

The results obtained in the present paper indicated that both AE and CC extracts are able to protect cells and tissues against oxidative damage acting both as radical scavenging as well as by increasing the antioxidant activity in the cell line model. Nevertheless, the optimal treatment concentration for the use in humans remains to be found in further clinical studies. Of note, we demonstrated that both agrimony and artichoke water extracts, containing near 8% of total polyphenols, increased antioxidant enzymes, catalase, and superoxide dismutase expression, in an environment forced to the oxidative stress (by adding H_2_O_2_ to the cell culture); furthermore, the extracts were able to protect the plasmid DNA against hydroxyl radical treatment. The *in vivo* study on AE extract demonstrated the capacity to also act on damaged skin, suggesting potential positive activity in wound repair. Differences in the behavior of the two extracts containing nearly the same amount of total polyphenols may be related to different phytochemical composition, being the AE characterized mainly by procyanidins and flavonol glycosides, while CC by chlorogenic acid derivatives.
